# Prevalence of the NTE_KPC_-I on IncF Plasmids Among Hypervirulent *Klebsiella pneumoniae* Isolates in Jiangxi Province, South China

**DOI:** 10.3389/fmicb.2021.622280

**Published:** 2021-06-21

**Authors:** Qi-Sen Huang, Wenjian Liao, Zhijuan Xiong, Dan Li, Fang-Ling Du, Tian-xin Xiang, DanDan Wei, La-Gen Wan, Yang Liu, Wei Zhang

**Affiliations:** ^1^Department of Clinical Microbiology, First Affiliated Hospital of Nanchang University, Nanchang University, Nanchang, China; ^2^Department of Respiratory and Critical Care, First Affiliated Hospital of Nanchang University, Nanchang University, Nanchang, China; ^3^Department of Infectious Disease, First Affiliated Hospital of Nanchang University, Nanchang University, Nanchang, China

**Keywords:** carbapenem resistance, hypervirulent *Klebsiella pneumoniae*, NTEKPC-2, IncFII-like plasmids, ST11

## Abstract

Infection caused by carbapenem-resistant hypervirulent Klebsiella pneumoniae (CR-hvKP) has become a tricky health care threat in China and KPC-2 enzyme is a main factor mediating resistance to carbapenems of *K. pneumoniae*. Here, we report the characterization of the genetic environment of the blaKPC-2 gene in CR-hvKP clinical isolates from South China. Forty-five non-duplicated CR-hvKP isolates collected in Jiangxi Province from 2018 to 2019 were analyzed. Each of them were multidrug-resistant due to the presence not only of blaKPC-2 gene but also of other resistance determinants, including Metallo-β-lactamases (NDM-1), extended-spectrum β-lactamases (TEM-1, CTX-M-14, SHV-1), and plasmid-mediated quinolone resistance determinants (qnrS, aac(6′)-Ib-cr). After plasmid analyses of PCR-based replicon typing (PBRT), mapping PCR, amplicon sequencing, and whole-genome sequencing (WGS) were used to analyze the genetic environment of the blaKPC-2 gene. PCR analysis of pLVPK-like plasmids, Southern Blot, and mouse lethality assay were used to characterize the virulence phenotype of *K. pneumoniae*. Multilocus sequence typing (MLST) analysis showed ST11 CR-hvKP was the predominant clone. In conclusion, this is the first analysis of diverse genetic structures blaKPC-2 gene in CR-hvKP isolates from south China. Both the NTEKPC-I on the IncF plasmids and pLVPK-like virulence plasmids make contributions to the formation of CR-hvKP especially ST11 which need more attention.

## Introduction

Recently, carbapenem-resistant hypervirulent *Klebsiella pneumoniae* (CR-hvKP) have become important pathogens of morbidity and mortality among hospital-acquired and long-term care-associated infections (Gu et al., 2018; Zhang et al., 2020). Regardless which plasmids-associated mechanisms underlying the formation of CR-hvKPs clones, the acquisition of carbapenem resistance plasmids by the hypervirulent *K. pneumoniae* (hvKP) strains or the acquisition of virulence plasmids by the carbapenem-resistant *K. pneumoniae* (CRKP) strains, carbapenem resistance plasmids are the main actors (Wyres et al., 2019).

KPC-2, the most common variant of KPC carbapenemase enzymes, is a main factor mediating resistance to carbapenems of *K. pneumoniae* (Shen et al., 2016). Transmission of the KPC gene, *bla*_*KPC*_, can be mediated by different molecular mechanisms such as the mobility of small genetic elements, horizontal transfer of plasmids, and the clonal spread (Munoz-Price and Quinn, 2009). In most countries and regions, such as Europe (Naas et al., 2008) and the United States (Chen et al., 2013), *bla*_*KPC*−2_ is mainly located on *Tn4401* transposon. However, *bla*_*KPC*_*-*bearing non-*Tn4401* elements (NTE_KPC_) were first reported on the plasmid pKP048 from a Chinese clinical *K. pneumoniae* isolate in 2009 (Shen et al., 2009). Then, NTE_KPC_ was not only reported in different provinces in China (Li et al., 2016; Wang et al., 2016; Fu et al., 2019), but also in other countries such as Brazil (Cerdeira et al., 2017, 2019) and Singapore (Octavia et al., 2019). In a review about molecular and genetic decoding in carbapenemase-producing *Klebsiella pneumoniae*, NTE_KPC_ has been divided into three groups (NTE_KPC_ I, NTE_KPC_ II, and NTE_KPC_ III) on the basis of the genes adjacent to *bla*_*KPC*_ (Chen et al., 2014).

The characteristics of this genetic structure, which mobilizes *bla*_*KPC*_ in CR-hvKP strains within and among different clones or plasmids, is unknown, and understanding it would provide insight into diffusion processes and their evolutionary history. This study aimed to present the genetic environment of *bla*_*KPC*−2_ in CR-hvKP isolates in Jiangxi Province using a series of PCR assays and whole-genome sequencing.

## Methods and Materials

### Bacterial Isolates and Antimicrobial Susceptibility Testing

A total of 45 non-duplicated CR-hvKP clinical isolates were collected from 11 prefecture-level cities in Jiangxi Province including Nanchang, Jingdezhen, Ganzhou, Jiujiang, Xinyu, Pingxiang, Yingtan, Ji'an, Yichun, Shangrao, and Fuzhou, from January 2018 to December 2019. Among them 38 CR-hvKP clinical isolates were collected from 513 non-duplicated CRKP clinical isolates in the First Affiliated Hospital of Nanchang University. *K. pneumoniae* isolates were identified by an automated Vitek II system (bioMerieux, Balmes-les-Grottes, France) and were further verified with 16S rRNA gene sequencing. According to the latest definition of hvKP (Xu et al., 2019; Zhang et al., 2020), we chose the CRKP strains carrying the pLVPK-like virulence plasmid as CR-hvKP strains in this study. Antibiotic susceptibilities were determined by the disk diffusion method on Mueller-Hinton agar according to the Clinical and Laboratory Standards Institute (CLSI) guidelines (Humphries et al., 2018). *K. pneumoniae* ATCC 700603 and *Escherichia coli* ATCC 25922 were used as quality control.

### PCR Detection of Resistance Genes, Virulence Genes, and Plasmid Replicon Types

Single PCR was used to analyze quinolone resistance genes [*aac(6*′*)-Ib-cr, qnrA, qnrB, qnrS*, and *qepA*], ESBLs genes (*bla*_*CTX*−*M*_, *bla*_*SHV*_*, bla*_*TEM*_), carbapenemase genes (*bla*_*KPC*_, *bla*_*NDM*_, *bla*_*OXA*−48_, *bla*_*IMP*_, *bla*_*VIM*_), and pLVPK-related loci (*rmpA, iutA, rmpA2*), as previously reported (Liao et al., 2020). To confirm the existence of the pLVPK-like plasmid, specific primers of *repA, sopB, Lv049* were also designed based on a previous study (Zhao et al., 2019). Plasmid incompatibility was analyzed by using PCR-based replicon typing (PBRT)-KIT 2.0 (DIATHEVA, Italy) (Zhou et al., 2020). A PCR mapping approach was carried out to compare the genetic context of the *bla*_*KPC*−2_ gene in all the CR-hvKP isolates with the *NTE*_*KPC*_ in plasmid pKP048 (Shen et al., 2009). All the PCR products were purified and sequenced, and their sequences were compared with the reference sequences stored in the GenBank nucleotide database.

### PFGE and MLST

S1 Nuclease Pulsed Field Gel Electrophoresis (S1-PFGE) and Southern blotting were used to determine the location of virulence genes. Hybridization were performed with the DIG-High Prime DNA Labeling and Detection Starter Kit II with of the probe *rmpA2* (Roche, Basel, Switzerland) (Li et al., 2020). All the CR-hvKP isolates were subjected to PFGE after digestion with XbaI. The molecular marker was *Salmonella* serotype *Braenderup* strain H9812. The cluster cutoff line at 80% similarity was used to analyze genetic relatedness. MLST of *Klebsiella pneumoniae* was performed online (http://bigsdb.pasteur.fr/klebsiella/klebsiella.html), as previously described (Liao et al., 2020).

### Whole-Genome Sequencing and Analysis

Given the PCR mapping analysis, we chose one isolate, respectively, from each different NTE_KPC_ pattern for WGS. Genome sequences were obtained using a combination of Illumina Miseq (150 bp paired-end), and they were assembled with SPAdes version 3.9.1. An average sequencing depth of ×64 was achieved for the genomes. Genomics analysis was performed as described in a previous study (Octavia et al., 2019).

### Nucleotide Sequence Accession Numbers

The genome sequences of 12 CR-hvKP strains were submitted to GenBank under the bioproject number PRJNA672246.

### Mouse Lethality Assay

Determination of the virulence of *K. pneumoniae* in mouse lethality tests and the medium lethal dose (LD50, expressed as colony-forming units) was performed as previously described (Yu et al., 2008). In short, a graded dose of 10^1^-10^7^ CFU of each strain in 10-fold serial dilutions in 0.1 ml of normal saline was injected intraperitoneally into mice (four mice for each dose of inoculum). The survival rate of all the vaccinated mice was recorded daily in 2 weeks. The hvKP strain NTUH-K2044 and the classic *K. pneumoniae* strain ATCC700603 were used as controls of high and low virulence strains, respectively. The interpretation of virulence was referred to reference (Siu et al., 2012).

### Ethics Statement

The study has been approved by the Ethics Committee of the First Affiliated Hospital of Nanchang University. Patients participating in the study were anonymous, as a result of the retrospective study, so informed consent was not obtained.

## Results

### Prevalence of ESBLs Genes and Quinolone Resistance Genes Among CR-hvKP Clinical Isolates

A total of 45 carbapenem-resistant hypervirulent *K. pneumoniae* isolates were selected from clinical specimens including 19 from sputum, 19 from blood, 4 from urine, 1 from pus, 1 from a deep vein catheter, and 1 from ascites. As shown in Supplementary Table 1, almost all the CR-hvKP isolates were resistant to 18 antibiotics commonly used in clinical treatment, except for some isolates that were sensitive to sulfamethoxazole, tobramycin, and amikacin. As shown in Table 1, all of the CR-hvKP isolates were found to carry carbapenemase gene *bla*_*KPC*−2_, three of which were found to co-carry carbapenemase gene *bla*_*NDM*−1_. Almost all of the CR-hvKP isolates were found to carry at least one ESBLs gene (*bla*_*CTX*−*M*_, *bla*_*SHV*_*, bla*_*TEM*_) and most of them were found to carry quinolone resistance gene *qnrS* and *aac(6*′*)-Ib-cr*.

**Table 1:** Main molecular features of all the CR-hvKP isolates.

**Isolates**	**LD50(cfu)**	**Virulence genes**	**Carbapenemase genes**	**Other durg resistance genes**	**NTE_**KPC**_ plasmid replicon type**
Kp1	5.4 × 10^4^	*rmpA, terW, silS, iutA, rmpA2*	*KPC-2*	*CTX-M-14, SHV-1, qnrS* *aac(6′)-Ib-cr*	IncFII(k)
Kp2	1.2 × 10^5^	*rmpA, terW, silS, iutA, rmpA2*	*KPC-2*	*CTX-M-14, qnrS* *aac(6′)-Ib-cr*	IncFII(k)
Kp3	3.7 × 10^4^	*rmpA, terW, silS, iutA, rmpA2*	*KPC-2*	*CTX-M-14, SHV-1, TEM-1, qnrS* *aac(6′)-Ib-cr*	–
Kp4	5.5 × 10^4^	*rmpA, terW, silS, iutA, rmpA2*	*KPC-2*	*CTX-M-14, SHV-1, TEM-1, qnrS* *aac(6′)-Ib-cr*	–
Kp5	1.1 × 10^5^	*rmpA, terW, silS, iutA, rmpA2*	*KPC-2*	*CTX-M-14, SHV-1, TEM-1, qnrS* *aac(6′)-Ib-cr*	IncFII(k)
Kp6	5.7 × 10^4^	*rmpA, terW, silS, iutA, rmpA2*	*KPC-2*	*CTX-M-14, SHV-1, TEM-1, qnrS* *aac(6′)-Ib-cr*	–
Kp7	3.2 × 10^5^	*rmpA, silS, iutA, rmpA2*	*KPC-2, NDM-1*	*CTX-M-14, SHV-1, TEM-1, qnrS*	IncFII(k)
Kp8	7.8 × 10^4^	*rmpA, terW, silS, iutA, rmpA2*	*KPC-2*	*CTX-M-14, SHV-1, TEM-1, qnrS* *aac(6′)-Ib-cr*	IncFII(k)
Kp9	7.7 × 10^5^	*rmpA, silS, iutA, rmpA2*	*NDM-1*	*CTX-M-14, SHV-1, TEM-1, qnrS* *aac(6′)-Ib-cr*	–
Kp10	8.1 × 10^5^	*rmpA, silS, iutA, rmpA2*	*KPC-2, NDM-1*	*CTX-M-14, SHV-1, TEM-1, qnrS* *aac(6′)-Ib-cr*	IncFII(k)
Kp11	2.1 × 10^4^	*rmpA, terW, silS, iutA, rmpA2*	*KPC-2*	*CTX-M-14, SHV-1, qnrS* *aac(6′)-Ib-cr*	IncFII(k)
Kp12	3.3 × 10^4^	*rmpA, terW, silS, iutA, rmpA2*	*KPC-2*	*CTX-M-14, TEM-1, qnrS* *aac(6′)-Ib-cr*	IncFII(k)
Kp13	6.5 × 10^3^	*rmpA, terW, silS, iutA, rmpA2*	*KPC-2*	*CTX-M-14, SHV-1, TEM-1, qnrS* *aac(6′)-Ib-cr*	IncFII(k)
Kp14	4.2 × 10^4^	*rmpA, terW, silS, iutA, rmpA2*	*KPC-2*	*CTX-M-14, TEM-1, qnrS* *aac(6′)-Ib-cr*	IncFII(k)
Kp15	7.9 × 10^5^	*terW, silS, iutA, rmpA2*	*KPC-2*	*CTX-M-14, TEM-1, qnrS* *aac(6′)-Ib-cr*	IncFII(k)
Kp16	7.6 × 10^4^	*rmpA, terW, silS, iutA, rmpA2*	*KPC-2*	*CTX-M-14, TEM-1, qnrS* *aac(6′)-Ib-cr*	IncH1B/IncF_repB_
Kp17	4.5 × 10^4^	*rmpA, terW, silS, iutA, rmpA2*	*KPC-2,VIM-2*	*CTX-M-14, SHV-1, TEM-1, qnrS* *aac(6′)-Ib-cr*	IncFII(k)
Kp18	8.5 × 10^5^	*terW, silS, rmpA2*	*KPC-2*	*CTX-M-14, SHV-1, TEM-1*	IncFII(k)
Kp19	7.7 × 10^3^	*rmpA, terW, silS, iutA, rmpA2*	*KPC-2*	*SHV-1, TEM-1, qnrS* *aac(6′)-Ib-cr*-	–
Kp20	7.7 × 10^4^	*terW, silS, iutA, rmpA2*	*KPC-2*	*SHV-1, TEM-1, qnrS* *aac(6′)-Ib-cr*-	–
Kp21	6.1 × 10^3^	*rmpA, terW, silS, iutA, rmpA2*	*KPC-2*	–	IncFII(k)
Kp22	6.4 × 10^5^	*terW, silS, iutA, rmpA2*	*KPC-2*	*CTX-M-14, SHV-1, TEM-1, qnrS*	IncFII(k)
Kp23	7.2 × 10^4^	*rmpA, terW, silS, iutA, rmpA2*	*KPC-2*	*CTX-M-14, SHV-1*	IncFII(k)
Kp24	8.8 × 10^5^	*terW, iutA, rmpA2*	*KPC-2*	*CTX-M-14, SHV-1, TEM-1*	IncFII(k)
Kp25	6.9 × 10^5^	*terW, silS, iutA, rmpA2*	*KPC-2*	*CTX-M-14, SHV-1, TEM-1*	IncFII(k)
Kp26	8.9 × 10^5^	*terW, iutA, rmpA2*	*KPC-2*	*CTX-M-14, SHV-1, TEM-1, qnrS*	IncFII(k)
Kp27	6.2 × 10^4^	*rmpA, terW, silS, iutA, rmpA2*	*KPC-2*	*CTX-M-14, SHV-1, TEM-1, qnrS*	IncFII(k)
Kp28	6.4 × 10^5^	*terW, silS, iutA, rmpA2*	*KPC-2*	*CTX-M-14, TEM-1, qnrS*	IncH1B/IncF_repB_
Kp29	5.7 × 10^4^	*rmpA, terW, silS, iutA, rmpA2*	*KPC-2*	*CTX-M-14, SHV-1, TEM-1, qnrS*	IncFII(k)
Kp30	6.2 × 10^4^	*rmpA, terW, silS, iutA, rmpA2*	*KPC-2*	*CTX-M-14, SHV-1, TEM-1, qnrS*	IncFII(k)
Kp31	3.8 × 10^4^	*rmpA, terW, silS, iutA, rmpA2*	*KPC-2*	*CTX-M-14, qnrS*	–
Kp32	3.9 × 10^5^	*terW, silS, iutA, rmpA2*	*KPC-2*	*CTX-M-14, SHV-1, TEM-1, qnrS*	–
Kp33	6.8 × 10^4^	*rmpA, terW, silS, iutA, rmpA2*	*KPC-2*	*CTX-M-14, SHV-1, qnrS*	IncFII(k)
Kp34	3.9 × 10^3^	*rmpA, terW, silS, iutA, rmpA2*	*KPC-2*	*TEM-1*	IncFII(k)
Kp35	6.3 × 10^4^	*rmpA, terW, silS, iutA, rmpA2*	*KPC-2*	*CTX-M-14, SHV-1, TEM-1, qnrS*	IncFII(k)
Kp36	8.3 × 10^4^	*rmpA, terW, silS, iutA, rmpA2*	*KPC-2*	*CTX-M-14, SHV-1, qnrS*	IncFII(k)
Kp37	8.7 × 10^4^	*rmpA, terW, silS, iutA, rmpA2*	*KPC-2*	*CTX-M-14, SHV-1, TEM-1*	–
Kp38	3.4 × 10^5^	*terW, silS, iutA, rmpA2*	*KPC-2*	*CTX-M-14, SHV-1, TEM-1, qnrS*	IncFII(k)
Kp39	4.7 × 10^4^	*rmpA, terW, silS, iutA, rmpA2*	*KPC-2*	*CTX-M-14, SHV-1, qnrS*	IncFII(k)
Kp40	5.6 × 10^4^	*rmpA, terW, silS, iutA, rmpA2*	*KPC-2*	*CTX-M-14, qnrS*	IncFII(k)
Kp41	2.6 × 10^4^	*rmpA, terW, silS, iutA, rmpA2*	*KPC-2*	*CTX-M-14, TEM-1, qnrS*	–
Kp42	1.4 × 10^5^	*terW, silS, iutA, rmpA2*	*KPC-2*	*CTX-M-14, TEM-1, qnrS*	IncFII(k)
Kp43	2.7 × 10^5^	*rmpA, terW, silS, rmpA2*	*KPC-2*	*SHV-1, qnrS*	–
Kp44	4.4 × 10^4^	*terW, silS, iutA, rmpA2*	*KPC-2*	*SHV-1, qnrS*	IncFII(k)
Kp45	2.9 × 10^5^	*rmpA, silS, iutA, rmpA2*	*KPC-2*	*CTX-M-14, TEM-1, qnrS*	IncFII(k)

### Virulence Assessment of CR-hvKP Clinical Isolates

In this scenario, PCR analysis of pLVPK-like plasmids revealed that all the CR-hvKP isolates were found to carry the pLVPK-like plasmid. These results were also verified by S1-PFGE and Southern blotting as shown in Supplementary Figure 1. However, PCR analysis of virulence genes revealed that not every isolate was found to carry the complete pLVPK-related loci (*rmpA, iutA, rmpA2*). The mouse lethality assay also proved that all the CR-hvKP isolates having the 50% lethal dose (LD50) of <10^5^ CFU were hypervirulent, and were a little lower virulent than the hvKP strain NTUH-K2044 having the LD50 of <10^2^ CFU, while classic *K. pneumoniae* strain ATCC700603 had the LD50 of more than 10^7^ CFU.

### Genetic Linkage of *bla_*kpc*−2_* Among the CR-hvKP Isolates

**Figure 1:**
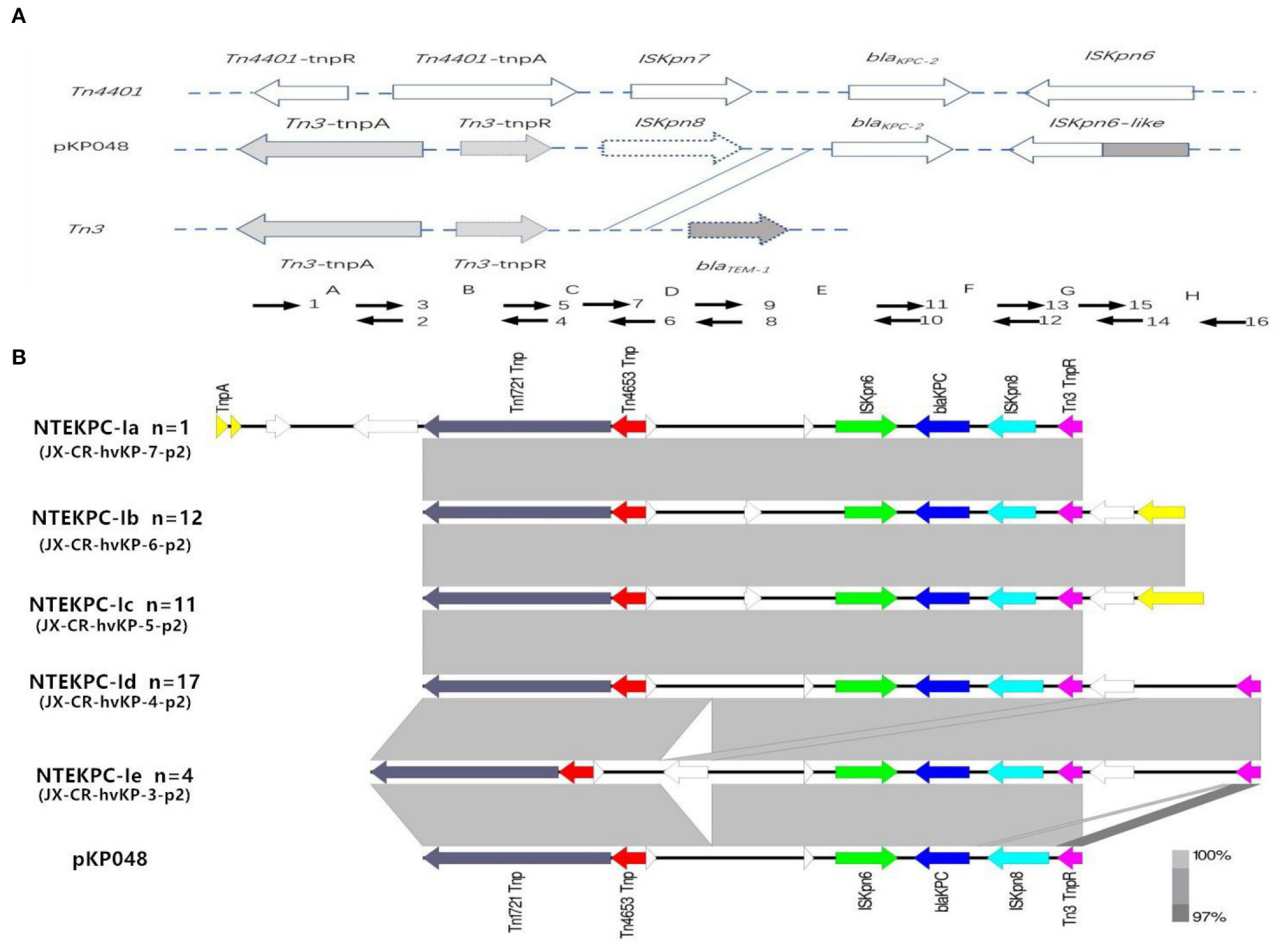
Schematic representation of the genetic structures surrounding the blaKPC-2 gene in the CR-hvKP isolates. **(A)** Classic *Tn3*-based structure in plasmid pKP048; Short arrowheads with numbers were used for PCR mapping. **(B)** Five distinct NTE patterns. Gene *bla*_*KPC*−2_ is represented by a blue arrow, and the remaining genes are color coded. NTEKPC-Ib, NTEKPC-Ic, and NTEKPC-Id were the most common genetic structures in the CR-hvKP isolates.

The analysis of the genetic environment of *bla*_*KPC*−2_ genes showed five distinct NTE patterns compared with the classic *Tn3*-based structure in the plasmid pKP048. All the different NTE patterns were classified as NTE_KPC_-I. In detail, the distribution of different NTE patterns among these clinical CR-hvKP strains was shown in the Figure 1.

### NTE_kpc_-I Plasmids Diversity

**Figure 2:**
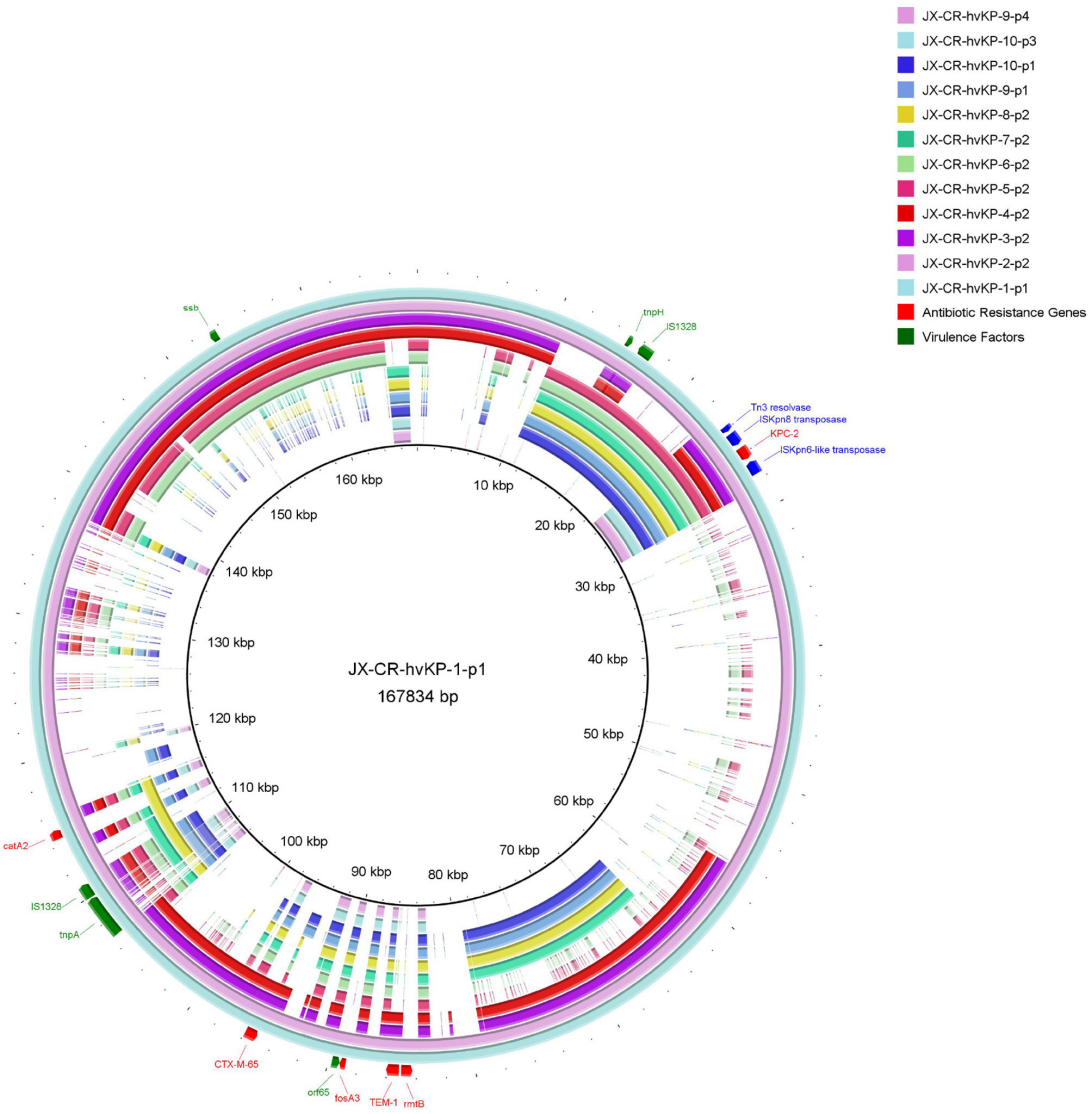
Gene map of twelve NTE_KPC_ plasmids harbored by CR-hvKP strains. JX-CR-hvKP-5-p2 and JX-CR-hvKP-6-p2 are from ST23 CR-hvKP, and the others are from ST11 CR-hvKP. The circular map was generated using the BLAST Ring Image Gnerator. A sequence comparison showed the polymorphism of *bla*_*KPC*_ plasmids in CR-hvKP.

Almost all the different NTE patterns carrying *blaKPC-2* were shown to be carried on the IncF plasmids. Interestingly untyped plasmid replicons were detected in 11 CR-hvKP isolates (Table 1). As shown in Figure 2, these NTE_KPC_ plasmids also had diversity of gene structure. Plasmid gene structure differences existed in the same ST (ST11) clinical *K. pneumoniae* strains. JX-CR-hvKP-9-p1 and JX-CR-hvKP-9-p4 from the same isolate had the totally different plasmid gene structure, the same goes for JX-CR-hvKP-10-p1 and JX-CRhvKP-10-p3. Interestingly JX-CR-hvKP-6-p2 and JX-CR-hvKP-5-p2 from ST23 CR-hvKP had the same plasmid gene structure, while JX-CR-hvKP-8-p2, JX-CR-hvKP-7-p2, JX-CR-hvKP-9-p1, JX-CR-hvKP-10-p1 from ST11 CR-hvKP also had highly similar plasmid gene structure.

### Molecular Characteristics

**Figure 3:**
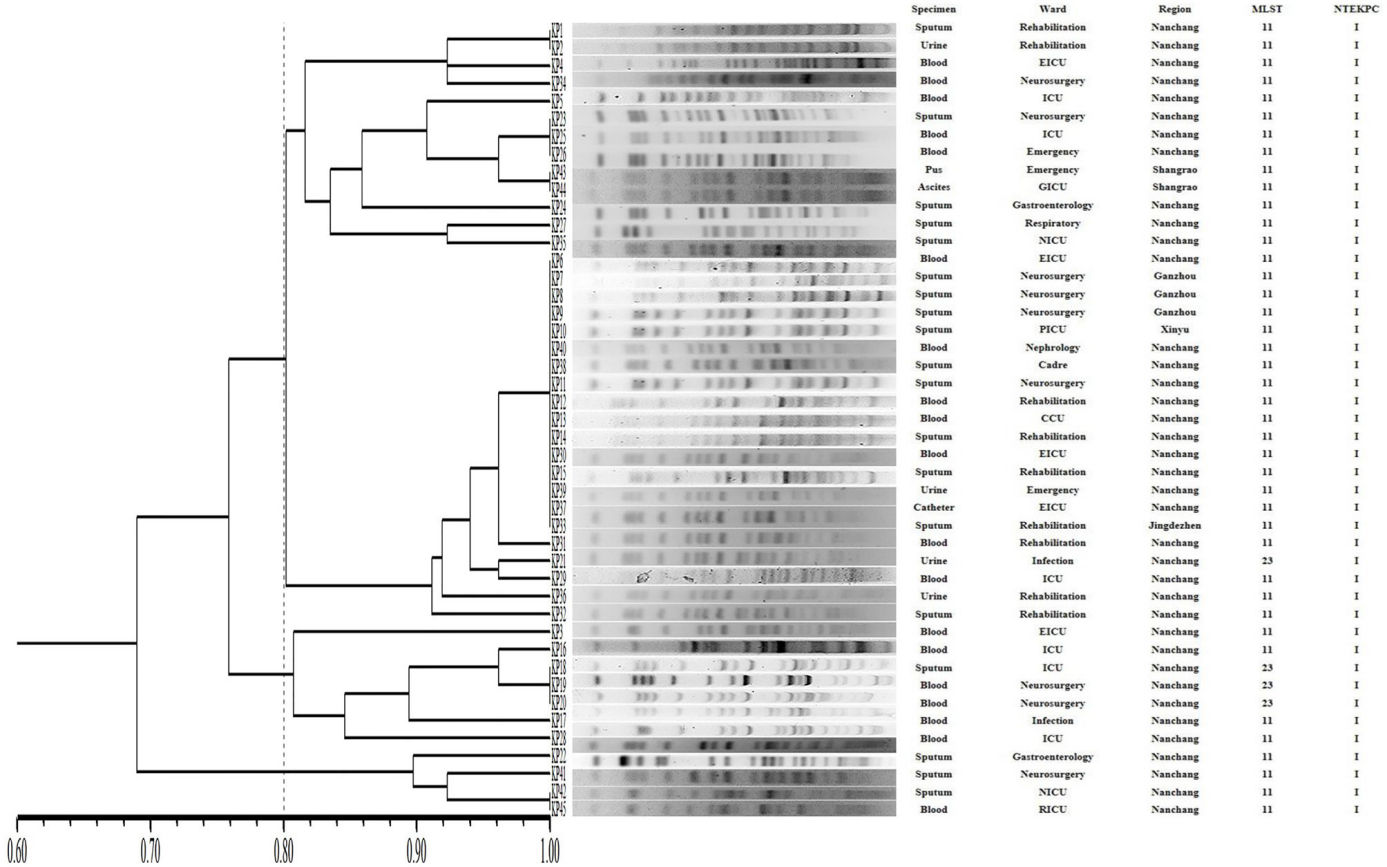
Pulsed-field gel electrophoresis (PFGE) patterns, NTE_KPC_ patterns, and STs among 45 clinical CR-hvKP isolates. NICU, neurosurgery ICU; GICU, gastroenterology ICU; RICU, respiratory ICU; PICU, Pediatric ICU; EICU, Emergency ICU.

The PFGE-based fingerprints of the CR-hvKP isolates displayed three different clusters (named A–C) using a similarity cutoff value of 80% (Figure 3), including cluster A (34/45, 75.6%), cluster B (7/45, 15.6%), and cluster C (4/45, 8.8%). The MLST analysis distinguished two different STs. The most prevalent ST in CR-hvKP isolates was ST11 (41/45, 91.1%), followed by ST23 (4/45, 8.9%).

## Discussion

Over the past few decades, hvKP has emerged worldwide, causing invasive infections since the first clinical hvKP report was published in 1986 (Russo and Marr, 2019). Although initially reported hvKP isolates were usually antimicrobial sensitive, clonal complexes of hypervirulent (hvKP) and carbapenem-resistant (CR) strains are non-overlapping (Liao et al., 2020). In this study, we collected 45 CR-hvKP strains causing nosocomial infections, including 41 ST11 CRKP acquiring the pLVPK like plasmid, 4 ST23 hvKP acquiring the carbapenemase plasmid. It was consistent with the evolution of CR-hvKP in a Chinese multicenter and molecular epidemiological analysis that the KPC-2-producing ST11 clone was the common type of CR-hvKP isolates (Zhang et al., 2020).

PCR analysis of *repA, sopB, Lv049* and Southern Blot revealed that all the CR-hvKP carrying pLVPK-like virulence plasmids were identified in this study. PCR analysis of pLVPK-related loci (*rmpA, iutA, rmpA2*) revealed that some virulence genes such as *rmpA, terW* were lost in a few CR-hvKP isolates. Maybe some virulence genes such as *rmpA, terW* were lost at the time of pLVPK-like plasmid transferring. It was consistent with the findings in a previous study about core genome allelic profiles of clinical *Klebsiella pneumoniae* strains based on multi-locus sequence typing scheme for hypervirulence analysis (Lan et al., 2020). Moreover, mouse lethality assay revealed that all the CR-hvKP isolates had a 50% lethal dose (LD50) of <10^5^ CFU, while classic *K. pneumoniae* (cKP) strain ATCC700603 had the LD50 of more than 10^7^ CFU. To our best knowledge, at present, the mouse lethality assay is the most standard method to differentiate hvKP from cKP (Russo and MacDonald, 2020).

This study first provides key insights into the horizontal transfer of the NTE_KPC_ and IncF plasmids, which appears to be a potential element driving the molecular diversification in ST11 CR-hvKP isolates. Transposon elements are believed to be responsible for the rapid spread of *bla*_*KPC*_ (Chen et al., 2011; Zhang et al., 2012). In China, a different genetic organization of the *bla*_*KPC*_ locus from the “traditional” Tn4401 was detected by Shen et al. (2009). The genetic locus located on the plasmid pKP048 contains a *Tn3*-based transposon and a partial Tn4401 segment, *ISKpn8*, and an *ISKpn6*-like element (Shen et al., 2009). Afterward, various mutations in the surrounding environment of the *bla*_*KPC*−2_ gene were detected, and most of which were mainly caused by the insertion of a truncated *bla*_*TEM*_ gene sequence between *ISKpn8* and *bla*_*KPC*_ gene with different sizes (Yang et al., 2013; Li et al., 2016). In this study, the KPC structures of all the CR-hvKP isolates should be separated into the NTE_KPC_-I group because of the *bla*_*TEM*_ absence (NTE_KPC_-I) (Figure 1) (Chen et al., 2014). PCR and WGS analysis showed that there were five different NTE_KPC_-I patterns in these CR-hvKP strains. They had high similarity to the NTE_KPC_ on the earliest plasmid pKP048. An increasing number of CR-hvKP in Jiangxi Province revealed that NTE_KPC_-I had strong dissemination ability and good stability (Liao et al., 2020; Li et al., 2020). It is noteworthy that NTE_KPC_s are primarily found in non-ST258 *K. pneumoniae* or other non-*K. pneumoniae* species (Chen et al., 2014).

The pandemic spread of *bla*_*KPC*−2_ among *Klebsiella pneumoniae* ST11 in China is mainly related to the horizontal transfer mediated by incompatibility group F (IncF) plasmids (Chi et al., 2019; Fu et al., 2019). PCR-based replicon type and representative strain WGS analysis revealed that almost all the different NTE patterns carrying *bla*_*KPC*−2_ in this study were also shown to be carried on the IncF plasmids. We suppose that there was also a close correlation between NTE_KPC_-I and IncF plasmids in CR-hvKP, whereby ST11 CR-hvKP is a seemingly good colonizer to capture IncF plasmids. The gene map of twelve NTE_KPC_ plasmids showed that there are gene structure differences among ST11 CR-hvKP strains in Jiangxi Province, which is consistent with the finding that the diversity of the plasmids of genetically related *K. pneumoniae* strains harboring the beta-lactamase gene *blaKPC-2* existed in the Netherlands from 2014 to 2019 (Hendrickx et al., 2020). Interestingly JX-CR-hvKP-6-p2 and JX-CR-hvKP-5-p2 from ST23 CR-hvKP had the same plasmid gene structure without any plasmid replicon type. It appeared to be that the NTE_KPC_ plasmids lost their plasmid replicon after entering ST23 hvKP, while JX-CR-hvKP-8-p2, JX-CR-hvKP-7-p2, JX-CR-hvKP-9-p1, and JX-CR-hvKP-10-p1 from ST11 CR-hvKP had highly similar plasmid gene structure. This indicates that the resistance/virulence hybrid plasmids in JX-CR-hvKP-9 and JX-CR-hvKP-10 formed by the fusion of NTE_KPC_ plasmids and pLVPK-like virulence plasmids. The hypothesis must be validated by further experiments.

The PFGE patterns show that all the CR-hvKP isolates were assigned to three clusters based on >80% pattern similarity. It is consistent with a previous study in China that the ST11 genomes were highly heterogeneous and clustered into at least three major lineages based on single nucleotide polymorphism (SNP) analysis (Dong et al., 2018).

The study has certain limitations, including its retrospective nature and a relatively small study population. Therefore, there may be selection bias, which limits the general application of study results to other areas. Consequently, a further study that includes more patients, especially for ST23 hvKp isolates, is needed.

## Conclusion

In conclusion, this is the first analysis of the diverse genetic structures of the *bla*_*KPC*−2_ gene in CR-hvKP isolates from South China. Both the NTE_KPC_-I on the IncF plasmids and pLVPK-like virulence plasmids make contributions to the formation of CR-hvKP, especially ST11, which need more attention.

## Data Availability Statement

The datasets presented in this study can be found in online repositories. The names of the repository/repositories and accession number(s) can be found at: GenBank, PRJNA672246.

## Ethics Statement

The animal study was reviewed and approved by the Ethics Committee of the First Affiliated Hospital of Nanchang University. Written informed consent was obtained from the individual(s) for the publication of any potentially identifiable images or data included in this article.

## Author Contributions

L-GW and T-xX did strain characterization and participated in manuscript writing. ZX and DL conceived the study and performed data analysis. WL did the whole-genome sequencing and comparative genomics and participated in manuscript writing. YL and DW wrote the paper. Q-SH collected the clinical data and analyzed the data. All authors contributed to the article and approved the submitted version.

## Conflict of Interest

The authors declare that the research was conducted in the absence of any commercial or financial relationships that could be construed as a potential conflict of interest.
