# Supplementary material for: Prevalence of the NTEKPC-I on IncF Plasmids Among Hypervirulent Klebsiella pneumoniae Isolates in Jiangxi Province, South China
Source: Front Microbiol. 2021 Jun 21;12:622280. doi: 10.3389/fmicb.2021.622280 (PMC8256152; doi:10.3389/fmicb.2021.622280)
Supplement: Supplementary file 1 [file Table_1.DOC]

A total of 45 nonduplicate CR-hvKP clinical isolates were collected from the First Affiliated Hospital of Nanchang University in the southeastern region of China. Using S1-PFGE and Southern hybridization, we select *K. pneumoniae* strains carrying the pLVPK-like virulence plasmid as hvKP strains. The marker gene of the virulence plasmid rmpA2 was hybridized to confirm the presence of the pLVPK-like virulence plasmid.


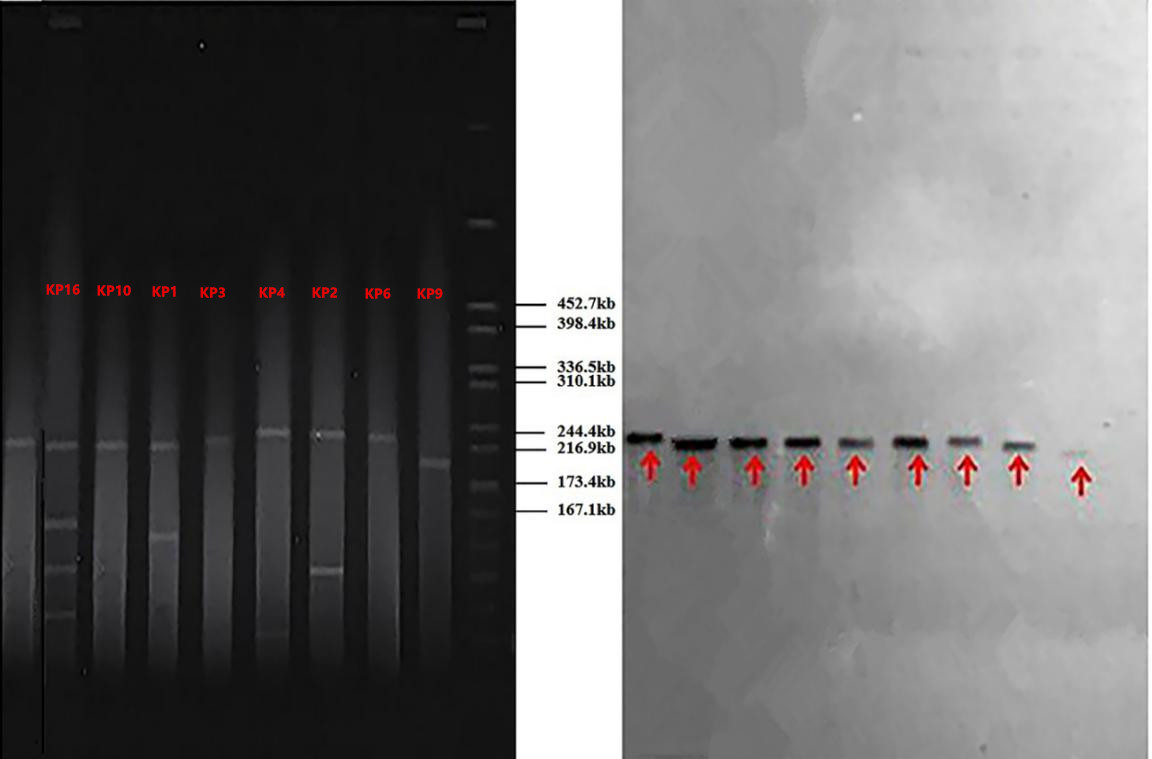


pLVPK-like plasmid

*rmpA2*

Supplementary Figure 1 S1-PFGE and Southern hybridisation of a portion of CR-hvKP strains in this study
