# Supplementary material for: Prevalence of the NTEKPC-I on IncF Plasmids Among Hypervirulent Klebsiella pneumoniae Isolates in Jiangxi Province, South China
Source: Front Microbiol. 2021 Jun 21;12:622280. doi: 10.3389/fmicb.2021.622280 (PMC8256152; doi:10.3389/fmicb.2021.622280)
Supplement: Supplementary file 2 [file Table_2.DOC]

Table1 Antibiotic resistance information

| Isolate  number | Levofloxacin | Imipenem | Tobramycin | Cefazolin | Cefoxitin | Ceftazidime | Cefotaxime | Cefoperazone Sulbactam | Cefepime | Gentamicin | Piperacillin tazobactam | Piperacillin | Meropenem | Ciprofloxacin | Sulfamethoxazole | Cefoperazone | Aztreonam | Amikacin |
| --- | --- | --- | --- | --- | --- | --- | --- | --- | --- | --- | --- | --- | --- | --- | --- | --- | --- | --- |
| Kp1 | R（>4ug/ml） | R  (>8ug/ml) | R  (>16ug/ml) | R  (>16ug/ml) | R  (>16ug/ml) | R  (>16ug/ml) | R  (>32ug/ml) | R  (>64ug/ml) | R  (>16ug/ml) | R  (>16ug/ml) | R  (>64ug/ml) | R  (>64ug/ml) | R  (>8ug/ml) | R  (>8ug/ml) | S  (<2ug/ml) | R  (>64ug/ml) | R  (>16ug/ml) | S  (<4ug/ml) |
| Kp2 | R  （>4ug/ml） | R  (>8ug/ml) | R  (>16ug/ml) | R  (>16ug/ml) | R  (>16ug/ml) | R  (>16ug/ml) | R  (>32ug/ml) | R  (>64ug/ml) | R  (>16ug/ml) | R  (>16ug/ml) | R  (>64ug/ml) | R  (>64ug/ml) | R  (>8ug/ml) | R  (>8ug/ml) | S  (<2ug/ml) | R  (>64ug/ml) | R  (>16ug/ml) | S  (<4ug/ml) |
| Kp3 | R  （>4ug/ml) | R  (>8ug/ml) | R  (>16ug/ml) | R  (>16ug/ml) | R  (>16ug/ml) | R  (>16ug/ml) | R  (>32ug/ml) | R  (>64ug/ml) | R  (>16ug/ml) | R  (>16ug/ml) | R  (>64ug/ml) | R  (>64ug/ml) | R  (>8ug/ml) | R  (>8ug/ml) | R  (>16ug/ml) | R  (>64ug/ml) | R  (>16ug/ml) | S  (<4ug/ml) |
| Kp4 | R  （>4ug/ml) | R  (>8ug/ml) | R  (>16ug/ml) | R  (>16ug/ml) | R  (>16ug/ml) | R  (>16ug/ml) | R  (>32ug/ml) | R  (>64ug/ml) | R  (>16ug/ml) | R  (>16ug/ml) | R  (>64ug/ml) | R  (>64ug/ml) | R  (>8ug/ml) | R  (>8ug/ml) | R  (>16ug/ml) | R  (>64ug/ml) | R  (>16ug/ml) | S  (<4ug/ml) |
| Kp5 | R  （>4ug/ml) | R  (>8ug/ml) | R  (>16ug/ml) | R  (>16ug/ml) | R  (>16ug/ml) | R  (>16ug/ml) | R  (>32ug/ml) | R  (>64ug/ml) | R  (>16ug/ml) | R  (>16ug/ml) | R  (>64ug/ml) | R  (>64ug/ml) | R  (>8ug/ml) | R  (>8ug/ml) | R  (>16ug/ml) | R  (>64ug/ml) | R  (>16ug/ml) | S  (<4ug/ml) |
| Kp6 | R  （>4ug/ml) | R  (>8ug/ml) | R  (>16ug/ml) | R  (>16ug/ml) | R  (>16ug/ml) | R  (>16ug/ml) | R  (>32ug/ml) | R  (>64ug/ml) | R  (>16ug/ml) | R  (>16ug/ml) | R  (>64ug/ml) | R  (>64ug/ml) | R  (>8ug/ml) | R  (>8ug/ml) | R  (>16ug/ml) | R  (>64ug/ml) | R  (>16ug/ml) | S  (<4ug/ml) |
| Kp7 | R  （>4ug/ml) | R  (>8ug/ml) | R  (>16ug/ml) | R  (>16ug/ml) | R  (>16ug/ml) | R  (>16ug/ml) | R  (>32ug/ml) | R  (>64ug/ml) | R  (>16ug/ml) | R  (>16ug/ml) | R  (>64ug/ml) | R  (>64ug/ml) | R  (>8ug/ml) | R  (>8ug/ml) | R  (>16ug/ml) | R  (>64ug/ml) | R  (>16ug/ml) | R  (>16ug/ml) |
| Kp8 | R  （>4ug/ml) | R  (>8ug/ml) | R  (>16ug/ml) | R  (>16ug/ml) | R  (>16ug/ml) | R  (>16ug/ml) | R  (>32ug/ml) | R  (>64ug/ml) | R  (>16ug/ml) | R  (>16ug/ml) | I  （32ug/ml) | R  (>64ug/ml) | R  (>8ug/ml) | R  (>8ug/ml) | R  (>16ug/ml) | R  (>64ug/ml) | R  (>16ug/ml) | R  (>16ug/ml) |
| Kp9 | R  （>4ug/ml) | R  (>8ug/ml) | R  (>16ug/ml) | R  (>16ug/ml) | R  (>16ug/ml) | R  (>16ug/ml) | R  (>32ug/ml) | R  (>64ug/ml) | R  (>16ug/ml) | R  (>16ug/ml) | R  (>64ug/ml) | R  (>64ug/ml) | R  (>8ug/ml) | R  (>8ug/ml) | R  (>16ug/ml) | R  (>64ug/ml) | R  (>16ug/ml) | R  (>16ug/ml) |
| Kp10 | R  （>4ug/ml) | R  (>8ug/ml) | R  (>16ug/ml) | R  (>16ug/ml) | R  (>16ug/ml) | R  (>16ug/ml) | R  (>32ug/ml) | R  (>64ug/ml) | R  (>16ug/ml) | R  (>16ug/ml) | R  (>64ug/ml) | R  (>64ug/ml) | R  (>8ug/ml) | R  (>8ug/ml) | R  (>16ug/ml) | S  (<16ug/ml) | R  (>16ug/ml) | R  (>16ug/ml) |
| Kp11 | R  （>4ug/ml) | R  (>8ug/ml) | R  (>16ug/ml) | R  (>16ug/ml) | R  (>16ug/ml) | R  (>16ug/ml) | R  (>32ug/ml) | R  (>64ug/ml) | R  (>16ug/ml) | R  (>16ug/ml) | R  (>64ug/ml) | R  (>64ug/ml) | R  (>8ug/ml) | R  (>8ug/ml) | S  (<2ug/ml) | R  (>64ug/ml) | R  (>16ug/ml) | R  (>16ug/ml) |
| Kp12 | R  （>4ug/ml) | R  (>8ug/ml) | R  (>16ug/ml) | R  (>16ug/ml) | R  (>16ug/ml) | R  (>16ug/ml) | R  (>32ug/ml) | R  (>64ug/ml) | R  (>16ug/ml) | R  (>16ug/ml) | R  (>64ug/ml) | R  (>64ug/ml) | R  (>8ug/ml) | R  (>8ug/ml) | S  (<2ug/ml) | R  (>64ug/ml) | R  (>16ug/ml) | R  (>16ug/ml) |
| Kp13 | R  （>4ug/ml) | R  (>8ug/ml) | R  (>16ug/ml) | R  (>16ug/ml) | R  (>16ug/ml) | R  (>16ug/ml) | R  (>32ug/ml) | R  (>64ug/ml) | R  (>16ug/ml) | R  (>16ug/ml) | R  (>64ug/ml) | R  (>64ug/ml) | R  (>8ug/ml) | R  (>8ug/ml) | R  (>16ug/ml) | R  (>64ug/ml) | R  (>16ug/ml) | R  (>16ug/ml) |
| Kp14 | R  （>4ug/ml) | R  (>8ug/ml) | R  (>16ug/ml) | R  (>16ug/ml) | R  (>16ug/ml) | R  (>16ug/ml) | R  (>32ug/ml) | R  (>64ug/ml) | R  (>16ug/ml) | R  (>16ug/ml) | R  (>64ug/ml) | R  (>64ug/ml) | R  (>8ug/ml) | R  (>8ug/ml) | R  (>16ug/ml) | R  (>64ug/ml) | R  (>16ug/ml) | R  (>16ug/ml) |
| Kp15 | R  （>4ug/ml) | R  (>8ug/ml) | R  (>16ug/ml) | R  (>16ug/ml) | R  (>16ug/ml) | R  (>16ug/ml) | R  (>32ug/ml) | R  (>64ug/ml) | R  (>16ug/ml) | R  (>16ug/ml) | R  (>64ug/ml) | R  (>64ug/ml) | R  (>8ug/ml) | R  (>8ug/ml) | R  (>16ug/ml) | R  (>64ug/ml) | R  (>16ug/ml) | R  (>16ug/ml) |
| Kp16 | R  （>4ug/ml) | R  (>8ug/ml) | R  (>16ug/ml) | R  (>16ug/ml) | R  (>16ug/ml) | R  (>16ug/ml) | R  (>32ug/ml) | R  (>64ug/ml) | R  (>16ug/ml) | R  (>16ug/ml) | R  (>64ug/ml) | R  (>64ug/ml) | R  (>8ug/ml) | R  (>8ug/ml) | S  (<2ug/ml) | R  (>64ug/ml) | R  (>16ug/ml) | R  (>16ug/ml) |
| Kp17 | R  （>4ug/ml) | R  (>8ug/ml) | R  (>16ug/ml) | R  (>16ug/ml) | R  (>16ug/ml) | R  (>16ug/ml) | R  (>32ug/ml) | R  (>64ug/ml) | R  (>16ug/ml) | R  (>16ug/ml) | R  (>64ug/ml) | R  (>64ug/ml) | R  (>8ug/ml) | R  (>8ug/ml) | S  (<2ug/ml) | R  (>64ug/ml) | R  (>16ug/ml) | R  (>16ug/ml) |
| Kp18 | S  (<2ug/ml) | R  (>8ug/ml) | R  (>16ug/ml) | R  (>16ug/ml) | R  (>16ug/ml) | R  (>16ug/ml) | R  (>32ug/ml) | R  (>64ug/ml) | I  (8ug/ml) | R  (>16ug/ml) | R  (>64ug/ml) | R  (>64ug/ml) | R  (>8ug/ml) | I  (4ug/ml) | S  (<2ug/ml) | R  (>64ug/ml) | R  (>16ug/ml) | R  (>16ug/ml) |
| Kp19 | S  (<2ug/ml) | R  (>8ug/ml) | S  (<4ug/ml) | R  (>16ug/ml) | R  (>16ug/ml) | S  (<4ug/ml) | R  (>32ug/ml) | I  (32ug/ml) | R  (>16ug/m) | R  (>16ug/ml) | R  (>64ug/ml) | R  (>64ug/ml) | R  (>8ug/ml) | I  (4ug/ml) | S  (<2ug/ml) | R  (>64ug/ml) | R  (>16ug/ml) | S  (<4ug/ml) |
| Kp20 | R  （>4ug/ml) | R  (>8ug/ml) | R  (>16ug/ml) | R  (>16ug/ml) | R  (>16ug/ml) | R  (>16ug/ml) | R  (>32ug/ml) | R  (>64ug/ml) | R  (>16ug/ml) | R  (>16ug/ml) | R  (>64ug/ml) | R  (>64ug/ml) | R  (>8ug/ml) | R  (>8ug/ml) | S  (<2ug/ml) | R  (>64ug/ml) | R  (>16ug/ml) | R  (>16ug/ml) |
| Kp21 | I  (2ug/ml) | R  (>8ug/ml) | S  (<4ug/ml) | R  (>16ug/ml) | R  (>16ug/ml) | R  (>16ug/ml) | I  (16ug/ml) | I  (32ug/ml) | R  (>16ug/ml) | R  (>16ug/ml) | R  (>64ug/ml) | R  (>64ug/ml) | S  (<4ug/ml) | R  (>8ug/ml) | R  (>16ug/ml) | R  (>64ug/ml) | R  (>16ug/ml) | S  (<4ug/ml) |
| Kp22 | S  (<2ug/ml) | R  (>8ug/ml) | S  (<4ug/ml) | R  (>16ug/ml) | R  (>16ug/ml) | R  (>16ug/ml) | R  (>32ug/ml) | I  (32ug/ml) | R  (>16ug/ml) | R  (>16ug/ml) | R  (>64ug/ml) | R  (>64ug/ml) | S  (<4ug/ml) | R  (>8ug/ml) | R  (>16ug/ml) | R  (>64ug/ml) | R  (>16ug/ml) | S  (<4ug/ml) |
| Kp23 | S  (<2ug/ml) | R  (>8ug/ml) | I  (8ug/ml) | R  (>16ug/ml) | R  (>16ug/ml) | R  (>16ug/ml) | R  (>32ug/ml) | R  (>64ug/ml) | R  (>16ug/ml) | R  (>16ug/ml) | R  (>64ug/ml) | R  (>64ug/ml) | S  (<4ug/ml) | R  (>8ug/ml) | S  (<2ug/ml) | R  (>64ug/ml) | R  (>16ug/ml) | S  (<4ug/ml) |
| Kp24 | I  (2ug/ml) | R  (>8ug/ml) | R  (>16ug/ml) | R  (>16ug/ml) | R  (>16ug/ml) | R  (>16ug/ml) | R  (>32ug/ml) | R  (>64ug/ml) | R  (>16ug/m) | R  (>16ug/ml) | R  (>64ug/ml) | R  (>64ug/ml) | R  (>8ug/ml) | I  (4ug/ml) | R  (>16ug/ml) | R  (>64ug/ml) | R  (>16ug/ml) | R  (>16ug/ml) |
| Kp25 | R  （>4ug/ml) | R  (>8ug/ml) | R  (>16ug/ml) | R  (>16ug/ml) | R  (>16ug/ml) | R  (>16ug/ml) | R  (>32ug/ml) | R  (>64ug/ml) | R  (>16ug/ml) | R  (>16ug/ml) | R  (>64ug/ml) | R  (>64ug/ml) | R  (>8ug/ml) | R  (>8ug/ml) | S  (<2ug/ml) | R  (>64ug/ml) | R  (>16ug/ml) | R  (>16ug/ml) |
| Kp26 | R  （>4ug/ml) | R  (>8ug/ml) | R  (>16ug/ml) | R  (>16ug/ml) | R  (>16ug/ml) | R  (>16ug/ml) | R  (>32ug/ml) | R  (>64ug/ml) | R  (>16ug/ml) | R  (>16ug/ml) | R  (>64ug/ml) | R  (>64ug/ml) | R  (>8ug/ml) | I  (4ug/ml) | R  (>16ug/ml) | R  (>64ug/ml) | R  (>16ug/ml) | R  (>16ug/ml) |
| Kp27 | I  (2ug/ml) | R  (>8ug/ml) | I  (8ug/ml) | R  (>16ug/ml) | R  (>16ug/ml) | R  (>16ug/ml) | R  (>32ug/ml) | R  (>64ug/ml) | R  (>16ug/ml) | R  (>16ug/ml) | I  （32ug/ml) | R  (>64ug/ml) | R  (>8ug/ml) | R  (>8ug/ml) | S  (<2ug/ml) | R  (>64ug/ml) | R  (>16ug/ml) | S  (<4ug/ml) |
| Kp28 | R  （>4ug/ml) | R  (>8ug/ml) | I  (8ug/ml) | R  (>16ug/ml) | R  (>16ug/ml) | R  (>16ug/ml) | R  (>32ug/ml) | I  (32ug/ml) | R  (>16ug/ml) | R  (>16ug/ml) | R  (>64ug/ml) | R  (>64ug/ml) | R  (>8ug/ml) | R  (>8ug/ml) | S  (<2ug/ml) | R  (>64ug/ml) | R  (>16ug/ml) | S  (<4ug/ml) |
| Kp29 | R  （>4ug/ml) | R  (>8ug/ml) | I  (8ug/ml) | R  (>16ug/ml) | R  (>16ug/ml) | R  (>16ug/ml) | I  (16ug/ml) | R  (>64ug/ml) | R  (>16ug/ml) | R  (>16ug/ml) | R  (>64ug/ml) | R  (>64ug/ml) | R  (>8ug/ml) | R  (>8ug/ml) | R  (>16ug/ml) | R  (>64ug/ml) | R  (>16ug/ml) | S  (<4ug/ml) |
| Kp30 | R  （>4ug/ml) | R  (>8ug/ml) | I  (8ug/ml) | R  (>16ug/ml) | R  (>16ug/ml) | R  (>16ug/ml) | I  (16ug/ml) | R  (>64ug/ml) | R  (>16ug/ml) | R  (>16ug/ml) | R  (>64ug/ml) | R  (>64ug/ml) | R  (>8ug/ml) | R  (>8ug/ml) | R  (>16ug/ml) | R  (>64ug/ml) | R  (>16ug/ml) | S  (<4ug/ml) |
| Kp31 | R  （>4ug/ml) | R  (>8ug/ml) | R  (>16ug/ml) | R  (>16ug/ml) | R  (>16ug/ml) | R  (>16ug/ml) | R  (>32ug/ml) | R  (>64ug/ml) | R  (>16ug/ml) | R  (>16ug/ml) | R  (>64ug/ml) | R  (>64ug/ml) | R  (>8ug/ml) | R  (>8ug/ml) | S  (<2ug/ml) | S  (<16ug/ml) | R  (>16ug/ml) | S  (<4ug/ml) |
| Kp32 | R  （>4ug/ml) | R  (>8ug/ml) | I  (8ug/ml) | R  (>16ug/ml) | R  (>16ug/ml) | R  (>16ug/ml) | R  (>32ug/ml) | R  (>64ug/ml) | R  (>16ug/ml) | R  (>16ug/ml) | R  (>64ug/ml) | R  (>64ug/ml) | R  (>8ug/ml) | R  (>8ug/ml) | R  (>16ug/ml) | R  (>64ug/ml) | R  (>16ug/ml) | S  (<4ug/ml) |
| Kp33 | R  （>4ug/ml) | R  (>8ug/ml) | R  (>16ug/ml) | R  (>16ug/ml) | R  (>16ug/ml) | R  (>16ug/ml) | R  (>32ug/ml) | I  (32ug/ml) | R  (>16ug/ml) | R  (>16ug/ml) | R  (>64ug/ml) | R  (>64ug/ml) | R  (>8ug/ml) | R  (>8ug/ml) | S  (<2ug/ml) | R  (>64ug/ml) | R  (>16ug/ml) | S  (<4ug/ml) |
| Kp34 | S  (<2ug/ml) | R  (>8ug/ml) | S  (<4ug/ml) | R  (>16ug/ml) | R  (>16ug/ml) | S  (<4ug/ml) | I  (16ug/ml) | S  (<16ug/ml) | S  (<4ug/ml) | R  (>16ug/ml) | R  (>64ug/ml) | R  (>64ug/ml) | R  (>8ug/ml) | S  (<2ug/ml) | S  (<2ug/ml) | R  (>64ug/ml) | R  (>16ug/ml) | S  (<4ug/ml) |
| Kp35 | R  （>4ug/ml) | R  (>8ug/ml) | R  (>16ug/ml) | R  (>16ug/ml) | R  (>16ug/ml) | R  (>16ug/ml) | R  (>32ug/ml) | R  (>64ug/ml) | R  (>16ug/ml) | R  (>16ug/ml) | R  (>64ug/ml) | R  (>64ug/ml) | R  (>8ug/ml) | R  (>8ug/ml) | S  (<2ug/ml) | R  (>64ug/ml) | R  (>16ug/ml) | R  (>16ug/ml) |
| Kp36 | R  （>4ug/ml) | R  (>8ug/ml) | R  (>16ug/ml) | R  (>16ug/ml) | R  (>16ug/ml) | R  (>16ug/ml) | R  (>32ug/ml) | I  (32ug/ml) | R  (>16ug/ml) | R  (>16ug/ml) | R  (>64ug/ml) | R  (>64ug/ml) | R  (>8ug/ml) | R  (>8ug/ml) | S  (<2ug/ml) | R  (>64ug/ml) | R  (>16ug/ml) | S  (<4ug/ml) |
| Kp37 | I  (2ug/ml) | R  (>8ug/ml) | S  (<4ug/ml) | R  (>16ug/ml) | R  (>16ug/ml) | R  (>16ug/ml) | R  (>32ug/ml) | I  (32ug/ml) | R  (>16ug/ml) | R  (>16ug/ml) | R  (>64ug/ml) | R  (>64ug/ml) | R  (>8ug/ml) | I  (4ug/ml) | S  (<2ug/ml) | R  (>64ug/ml) | R  (>16ug/ml) | S  (<4ug/ml) |
| Kp38 | R  （>4ug/ml) | R  (>8ug/ml) | S  (<4ug/ml) | R  (>16ug/ml) | R  (>16ug/ml) | R  (>16ug/ml) | R  (>32ug/ml) | R  (>64ug/ml) | R  (>16ug/ml) | R  (>16ug/ml) | R  (>64ug/ml) | R  (>64ug/ml) | R  (>8ug/ml) | R  (>8ug/ml) | R  (>16ug/ml) | R  (>64ug/ml) | R  (>16ug/ml) | S  (<4ug/ml) |
| Kp39 | R  （>4ug/ml) | R  (>8ug/ml) | S  (<4ug/ml) | R  (>16ug/ml) | R  (>16ug/ml) | R  (>16ug/ml) | R  (>32ug/ml) | I  (32ug/ml) | R  (>16ug/ml) | R  (>16ug/ml) | R  (>64ug/ml) | R  (>64ug/ml) | R  (>8ug/ml) | R  (>8ug/ml) | R  (>16ug/ml) | R  (>64ug/ml) | R  (>16ug/ml) | R  (>16ug/ml) |
| Kp40 | R  （>4ug/ml) | R  (>8ug/ml) | S  (<4ug/ml) | R  (>16ug/ml) | R  (>16ug/ml) | R  (>16ug/ml) | R  (>32ug/ml) | I  (32ug/ml) | R  (>16ug/ml) | R  (>16ug/ml) | R  (>64ug/ml) | R  (>64ug/ml) | R  (>8ug/ml) | R  (>8ug/ml) | S  (<2ug/ml) | R  (>64ug/ml) | R  (>16ug/ml) | S  (<4ug/ml) |
| Kp41 | R  （>4ug/ml) | R  (>8ug/ml) | I  (8ug/ml) | R  (>16ug/ml) | R  (>16ug/ml) | R  (>16ug/ml) | R  (>32ug/ml) | R  (>64ug/ml) | R  (>16ug/ml) | R  (>16ug/ml) | R  (>64ug/ml) | R  (>64ug/ml) | R  (>8ug/ml) | R  (>8ug/ml) | R  (>16ug/ml) | R  (>64ug/ml) | R  (>16ug/ml) | S  (<4ug/ml) |
| Kp42 | R  （>4ug/ml) | R  (>8ug/ml) | R  (>16ug/ml) | R  (>16ug/ml) | R  (>16ug/ml) | R  (>16ug/ml) | R  (>32ug/ml) | R  (>64ug/ml) | R  (>16ug/ml) | R  (>16ug/ml) | R  (>64ug/ml) | R  (>64ug/ml) | R  (>8ug/ml) | R  (>8ug/ml) | R  (>16ug/ml) | R  (>64ug/ml) | R  (>16ug/ml) | R  (>16ug/ml) |
| Kp43 | R  （>4ug/ml) | R  (>8ug/ml) | S  (<4ug/ml) | R  (>16ug/ml) | R  (>16ug/ml) | R  (>16ug/ml) | R  (>32ug/ml) | I  (32ug/ml) | R  (>16ug/ml) | R  (>16ug/ml) | R  (>64ug/ml) | R  (>64ug/ml) | R  (>8ug/ml) | R  (>8ug/ml) | R  (>16ug/ml) | R  (>64ug/ml) | R  (>16ug/ml) | S  (<4ug/ml) |
| Kp44 | R  （>4ug/ml) | R  (>8ug/ml) | I  (8ug/ml) | R  (>16ug/ml) | R  (>16ug/ml) | R  (>16ug/ml) | R  (>32ug/ml) | I  (32ug/ml) | R  (>16ug/ml) | R  (>16ug/ml) | R  (>64ug/ml) | R  (>64ug/ml) | R  (>8ug/ml) | R  (>8ug/ml) | R  (>16ug/ml) | R  (>64ug/ml) | R  (>16ug/ml) | S  (<4ug/ml) |
| Kp45 | R  （>4ug/ml) | R  (>8ug/ml) | R  (>16ug/ml) | R  (>16ug/ml) | R  (>16ug/ml) | R  (>16ug/ml) | R  (>32ug/ml) | R  (>64ug/ml) | R  (>16ug/ml) | R  (>16ug/ml) | R  (>64ug/ml) | R  (>64ug/ml) | R  (>8ug/ml) | R  (>8ug/ml) | R  (>16ug/ml) | R  (>64ug/ml) | R  (>16ug/ml) | R  (>16ug/ml) |
